# Performance Optimization and Characterization of Soda Residue-Fly Ash Geopolymer Paste for Goaf Backfill: Beta-Hemihydrate Gypsum Alternative to Sodium Silicate

**DOI:** 10.3390/ma13245604

**Published:** 2020-12-08

**Authors:** Haoyu Wang, Xianhui Zhao, Boyu Zhou, Yonghui Lin, Han Gao

**Affiliations:** 1 Tianjin University Renai College, Tianjin 301636, China; wanghaoyu1015@126.com (H.W.); gaohantianjin@163.com (H.G.); 2 School of Civil Engineering, Hebei University of Engineering, Handan 056038, China; 3 School of Civil and Transportation Engineering, Hebei University of Technology, Tianjin 300401, China; 201911601009@stu.hebut.edu.cn (B.Z.); 201611601012@stu.hebut.edu.cn (Y.L.); 4 Department of Economics and Management, Hebei Normal University for Nationalities, Chengde 067000, China

**Keywords:** paste backfill, geopolymer, alternatives, soda residue, microstructure, gel product

## Abstract

Solid waste soda residue (SR), as an industrial pollutant of water, air and soil environment, can be utilized to prepare the low-calcium fly ash (FFA)-based geopolymer paste activated by sodium silicate (NS) solution for goaf backfill. However, the high addition of NS produces the high cost and high strength of synthesized backfill material in the previous study. The objective of this research is to investigate the cost optimization method and performance evaluation of SR-FFA-based geopolymer backfill paste. The alkaline beta-hemihydrate gypsum (BHG) alternative to partial NS was proposed. Scanning electron microscopy (SEM), X-ray diffraction (XRD) as well as Fourier transform infrared spectrometer (FTIR) tests were performed to clarify the role of BHG and evaluate the microstructures and products of backfill pastes. The results show that 10% BHG alternative ratios effectively improve fluidity, setting time and compressive strength to satisfy the performance requirement of goaf backfill material. The gel products in the optimal backfill paste C4 with 10% BHG alternative ratios are determined as the coexistence of C-S-H gel, (N,C)-A-S-H gel and CaSO_4_·2H_2_O at 28 d. The research results can make extensive utilization of SR and FFA in cemented paste backfill to synthesize cleaner material at a larger scale.

## 1. Introduction

Geopolymer, as a potential substitution of cement material, has obtained worldwide and rapid development in recent years [1,2,3,4,5]. Geopolymer possesses similar and/or even superior mechanical and durable performances as OPC (ordinary Portland cement), such as high strength, high hardness, high chemical-resistance and low carbon, etc. [6,7,8]. Moreover, geopolymer has the potential to reduce CO_2_ emissions and energy consumption from OPC production by nearly 80% and 60%, respectively [9,10]. As an eco-friendly material, geopolymer is usually prepared using locally available by-products (such as fly ash, soda residue, red mud and blast furnace slag, etc.) [11,12,13]. Fly ash, as one of the typical by-products, is widely used to manufacture geopolymer material. Fly ash is commonly available (annually more than 500 million tons are produced in China) [14,15]. Therefore, manufacturing fly ash-based geopolymer is significant for promoting cleaner production of solid wastes.

Generally, low-calcium fly ash based geopolymer (FFA-GEO) has been restricted to high-strength precast construction materials at room temperature [16]. High-calcium fly ash (CaO > 10 wt.%) is alkali-activated to obtain good hardening and high strength owing to the high reactive effect [17]. However, low-calcium fly ash is alkali-activated to prepare geopolymer material that usually possesses the disadvantage of longer setting time and lower strength, and evenly 28 d compressive strength of FFA-GEO is lower than 10 MPa cured at room temperature [18,19,20]. However, the improved FFA-GEO paste incorporating calcium additives can be utilized well as goaf backfill materials [19,21]. For goaf backfill, the additions of calcium-sources or calcium-additives are the effective and direct methods to optimize and improve the fresh and mechanical performances of geopolymer backfill paste [22,23].

Soda residue (SR), composed of various Ca-containing components, is the by-product of the industrial Na_2_CO_3_ process. Just from three main coastal Na_2_CO_3_-enterprises in Tangshan, Lianyungang and Weifang in China, 0.93 million tons of SR are produced each year [24]. The SR threatens the local marine and local soil ecological environment through seepage [25]. To achieve the scientific recycling utilization of SR, it is used as a calcium source in FFA-GEO to prepare backfill grouting materials [24]. In a previous study, the designed optimal FFA-GEO paste SFN6 possesses fluidity of 260.0 mm, 28 d compressive strength of 3.70 MPa and stone ratio of 98.6%, which has high addition of activators up to 26.53% [24]. Then the mass mixing proportion (2:3 in SR-FA ratio of, 2.0 mol/L in solution concentration, and 1.2 in a liquid–solid ratio) of SFN6 leads to a high cost owing to 26.53% activators. Therefore, considering the need for better industrial utilization of FFA-GEO paste incorporating SR, it requires further cost-reduction investigation and properties’ optimization.

The main aim of this paper is to propose an optimization method (BHG alternatives to partial NS) to reduce cost and improve properties, and to evaluate the macro/micro properties of SR-FFA-based geopolymer backfill grouting paste. The geopolymer pastes with different BHG alternative ratios were designed to investigate the effect of BHG in backfill materials through fresh and mechanical properties. Furthermore, the microstructure and gel product were characterized by a series of microscopic tests (scanning electron microscopy (SEM), X-ray diffraction (XRD) and Fourier transform infrared (FTIR) spectroscopy tests) to investigate the reaction mechanisms. The research results provide an experimental basis for the resource and scientific utilization of solid waste (SR and FFA) at a larger scale.

## 2. Materials and Methods

### 2.1. Materials

Geopolymer backfill pastes are obtained from a chemical geosynthetics process using alkali-activated silica and alumina phase materials [3]. In this study, the raw materials used to manufacture geopolymer backfill paste are low-calcium fly ash (FFA), soda residue (SR), sodium silicate (NS) pellets and beta-hemihydrate gypsum (BHG) powder.

**Figure 1:**
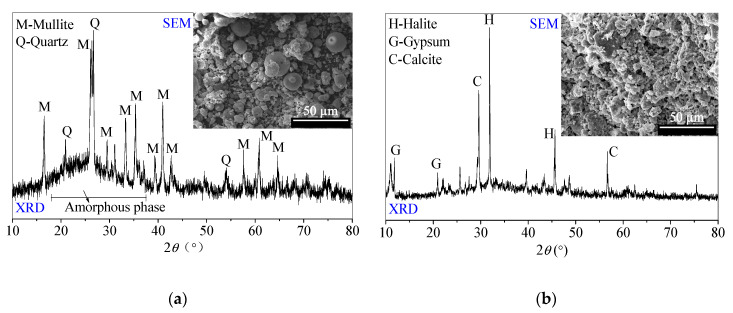
Scanning electron microscopy (SEM) photos and X-ray diffraction (XRD) patterns of (**a**) low-calcium fly ash (FFA) and (**b**) soda residue (SR) [24].

The chemical compositions and basic physical specifications of FFA and SR are listed in Table 1 and Table 2, respectively. XRD patterns and SEM images of SR and FFA are shown in Figure 1. The chemical and physical properties are consistent with those in the reported literature [3,23,24].

#### 2.1.1. Fly Ash (FFA)

The FFA, supported by a power plant in Tangshan city, Hebei Province (North of China), mainly consists of spherical glass microspheres with potential reactivity. Additionally, FFA is mainly composed of 48.37% SiO_2_ and 28.90% Al_2_O_3_. Besides crystal quartz and crystal mullite, the amorphous substances corresponding to the ‘hump’ appear at the range of 19–29° 2θ in the XRD pattern [24].

#### 2.1.2. Soda Residue (SR)

The SR is supported by a Na_2_CO_3_-production enterprise in Tangshan city, Hebei Province (North of China). The SR includes the various calcium-containing components, such as CaCO_3_, Ca (OH)_2_, CaCl_2_ and CaSO_4_. The SR usually possesses porous and loose micromorphology. Moreover, the SR includes gypsum, halite and calcite crystal phases in the XRD pattern. After being oven-dried at 40 °C, the SR is ground and sieved for less than 0.5 mm particle sizes for use, which is recommended by [24].

#### 2.1.3. Sodium Silicate (NS)

The NS pellets (analytical reagent and its selling price of 11.6–16.4 yuan/kg, SiO_2_/Na_2_O = 1.0, Na_2_SiO_3_·9H_2_O), as alkaline activators, were dissolved in distilled water to prepare the NS solution [26,27]. The NS solutions were allowed to cool to room temperature prior to prepare geopolymer backfill pastes.

#### 2.1.4. Beta-Hemihydrate Gypsum (BHG)

The BHG powder is produced by Xueli Building Material Company in Tianjin of China. Hemihydrate gypsum (Beta-type), expressed as CaSO_4_·0.5H_2_O and also called building gypsum, possesses slight expansion characteristics. Besides, the density of BHG is 2.60 g/cm^3^, specific surface area is 4300 cm^2^/g and pH value of BHG measures 12.48 at 100% water content. The BHG powder was utilized to synthesize the backfill paste without any pretreatment.

### 2.2. Preparation of Pastes

The geopolymer backfill pastes were manufactured using FFA, SR, BHG powder and NS solution. The NS pellets were first dissolved in the required water and settled until still, and then cooled to room temperature for use (NS solution, labelled as NSS). Subsequently, the designed NSS was added into the mixtures of FFA, SR and BHG powders to prepare the fresh pastes by stirring for two minutes to make them uniform, and one-step mixing which was regarded as a better mixing technique [24]. Then, the fresh pastes and controls were cast into Ø36 mm diameter cylindrical PVC moulds. The designed fresh pastes and controls were vibrated and then cured at the condition of 20 ± 2 °C temperature and 100% humidity. In particular, due to the lower hardening rate of alkali-activated FFA system [24], all of the samples were demoulded after cured for 7 d at the designed condition. After being demoulded, the samples continued to cure for 28 d under identical conditions.

In the study, a total of 25 groups of samples were prepared with different initial Ca/Si ratios and water contents (Table 3 and Table 4). Based on the reaction mechanism of SR-FFA-NS system backfill grouting material, the C–S–H gels generated from the reaction between SR and NS determined the early strength of the paste [24]. Thus, 18 groups of samples were used to clarify the relationships between 7 d unconfined compressive strength (UCS) and initial Ca/Si ratios (Table 3). Initial Ca/Si ratio means the molar ratio of CaCl_2_, Ca (OH)_2_ and CaSO_4_ in SR to NS. Additionally, seven groups of samples were utilized to explore the influence of water content on workability and early strength (Table 4). Furthermore, to determine the effect of BHG alternatives on the fresh and mechanical strength of FFA-GEO backfill pastes, nine groups of paste were prepared by a one-step mixing technique (Table 5).

### 2.3. Testing Methods

#### 2.3.1. Determination of Fresh and Hardened Properties

The workability and mechanical strength were measured to determine the optimal mixing proportion of hardened backfill pastes, which included fluidity, initial setting time, final setting time, stone rate and water-separation rate of fresh pastes as well as UCS. For all of the tests, the results were the averaged value from five identical samples.

(1) Measurement of fluidity and setting time

Fluidity demonstrates the workability of backfill grouting paste and determines whether the grouting materials can be successfully pumped into goaf [28]. The slump cone was used to measure the fluidity as a method reported in the literature [29]. Moreover, setting times are utilized to characterize the time when the backfill grouting paste starts losing plasticity (called as initial setting times), and the time when plasticity completely disappears and strength starts (called final setting times). The initial and final setting times were measured with the Vicat apparatus in accordance with the GB/T 1346-2011 standard (China) [19,24].

(2) Measurement of stone rate and water-separation rate

The stone rate and water-separation rate illustrate the backfill effects of pastes in the grouting engineering. Both of the performance indicators were determined by the tubes in accordance with GB/T 1346-2011 standard (China) and the method reported in the literature [24].

(3) Determination of unconfined compressive strength (UCS)

The UCS, as an important mechanical property, demonstrates whether the backfill grouting materials are damaged under loading [30]. The cylindrical samples with height-diameter ratio 2:1 (36.0 mm in diameter and 72.0 mm in height [21]) were prepared for measurement. The UCSs of hardened pastes and controls were tested at 7 d and 28 d by a compressing machine with the loading strain rates of 1.0 mm/min [19]. In particular, the solidification characteristics of pastes and controls were observed before failure.

#### 2.3.2. Micro-Characteristics of Hardened Backfill Pastes

After the determination of optimal backfill pastes for the fresh and hardened properties, the hardened paste C4, control SN5 and control C0 at 7 d and 28 d were further detected. Their gel product compositions and reaction mechanisms were verified through a series of micro-testing techniques (SEM, XRD and FTIR).

SEM test: The morphologies of the samples SN5, C0 and C4 were detected at 28 d. A Quanta FEG450 scanning electron microscope (Hillsboro, OR, USA) was employed.

XRD test: The XRD specimens were taken from the samples SN5, C0 and C4 at 7d and 28 d. Rigaku D/MAX-2500 X-ray diffraction spectroscopy with CuKα radiation (Akishima, Tokyo, Japan) was employed to detect the mineral compositions, using the scanning rate of 2°/min from 10° to 80° 2θ.

FTIR test: The samples SN5, C0 and C4 at 7d and 28 d were weighed into 1.3 ± 0.001 mg together with 130 mg KBr pellets to make tested specimens. A Nexus 8 Fourier transformation infrared spectroscopy (Bruker, Karlsruhe, Germany) was utilized to obtain FTIR spectra with the wavenumbers from 400 cm^−1^ to 3800 cm^−1^, and show the changes in the characteristic vibration peaks of Si-O-T (Si or Al) bonds.

## 3. Results and Discussion

### 3.1. Influence of Initial Ca/Si Ratios on Fluidity and 7 d Unconfined Compressive Strength (UCS)

For SR-FFA-based geopolymer backfill paste, in the previous study it was revealed that the inorganic calcium-containing salts (CaCl_2_, Ca(OH)_2_ and CaSO_4_) in SR reacted with NS to form C-S-H gels that mainly controlled the early strength; and while FFA was activated by NS to form (N,C)–A–S–H gels to enhance the later strength [24]. Therefore, the gel products in SR-FFA based geopolymer backfill paste activated by NS were verified as mixtures of C–S–H gels and (N,C)–A–S–H gels. The reaction mechanisms of C–S–H gels were expressed as the following formulae (1), (2) and (3). *n* refers to any reaction coefficient [24]. In particular, the properties of C-S-H gels are closely related to its initial Ca/Si ratios in raw materials.

CaCl_2_ + Na_2_SiO_3_ + *n*H_2_O → CaSiO_3_·*n*H_2_O↓ + 2NaCl (1)

Ca(OH)_2_ + Na_2_SiO_3_ + *n*H_2_O → CaSiO_3_·*n*H_2_O↓ + 2NaOH (2)

CaSO_4_ + Na_2_SiO_3_ + *n*H_2_O → CaSiO_3_·*n*H_2_O↓ + Na_2_SO_4_ (3)

Based on the geopolymeric process proposed by Hua et al. [31,32], the reactions of (N,C)-A-S-H gels occur in alkaline environment as follows (4) and (5) [24]. *n* is any reaction coefficient.

*n*(Si_2_O_5_, Al_2_O_3_) + 2*n*SiO_2_ + 4*n*H_2_O + (Na^+^, Ca^2+^) + OH^−^ → (Na^+^, Ca^2+^) + *n*(OH)_3_-Si–O–Al^−^(OH)_2_–O–Si–(OH)_3_ (4)

*n*(OH)_3_-Si–O–Al^−^(OH)_2_–O–Si–(OH)_3_ + (Na^+^, Ca^2+^) + OH^−^ → (Na^+^, Ca^2+^)-[Si(OH)_2_–O-Al^−^(OH)_2_–O–Si–(OH)_3_–O–]*_n_*+ 4*n*H_2_O (5)

In summary, it is significant to clarify the influence of initial Ca/Si ratio on early strength and solidification, which determines early workability of fresh backfill paste.

**Figure 2:**
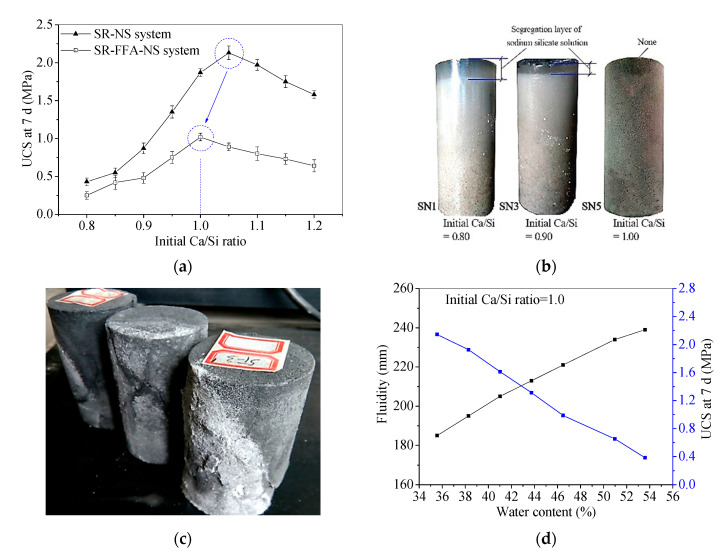
(**a**) Unconfined compressive strength (UCS) at 7 d, (**b**) solidification characteristics vs. Initial Ca/Si ratio for hardened pastes, (**c**) pan-alkali for hardened backfill paste, and (**d**) fluidity and UCS at 7 d vs. water contents.

The fluidity and 7 d UCS results for the SR-NS system and SR-FFA-NS system samples with different initial Ca/Si ratios are shown in Figure 2. The 7 d UCS increases first and then decreases with the initial Ca/Si ratios. The peak behaves at the range of 1.0~1.1 in initial Ca/Si ratios for SR-NS system samples, and while the maximum UCS appears at 1.0 in initial Ca/Si ratio for SR-FFA-NS system samples (Figure 2a). The small offset from 1.1 to 1.0 in initial Ca/Si ratio is attributed to the addition of FFA. This may be because that FFA is activated by NS to produce (N,C)–A–S–H gels together with some Ca^2+^ cations. Additionally, the segregation layer of NS appears at 0.8 and 0.9 in an initial Ca/Si ratio for SR-NS system pastes, and better solidification behaves at 1.0 in initial Ca/Si ratio (Figure 2b). In particular, the experimental result of the SR-FFA-NS system pastes shows the same phenomenon as the SR-NS system pastes. Thus, it is revealed that the higher 7 d UCS and the better solidification effect obtain at nearly 1.0 of initial Ca/Si ratio for SR-NS system and SR-FFA-NS system pastes.

In a previous study, the C–S–H gels generated in SR-FFA-NS system pastes produced cementation to determine the early strength [24]. If the effective components CaCl_2_, Ca(OH)_2_ and CaSO_4_ in SR react with NS at the initial Ca/Si ratio of 1.0, it leads to the highest contents of C–S–H gels and the highest values of 7 d UCS. However, the backfill paste (2:3 in SR-FA ratio, 2.0 mol/L in NS solution concentration and 1.2 in liquid-solid ratio) reported in the previous study was calculated as initial Ca/Si ratio 0.845 (lower than 1.0) [24], and was there evenly is the pan-alkali for hardened backfill paste (Figure 2c), which presents the excessive NS for SR-FFA based geopolymer backfill pastes. Therefore, the mass mixing proportion of alkaline activator NS should be further optimized to promote wider industrial application in the backfill materials.

Furthermore, the 7 d UCS of the SR-FFA-NS system pastes with initial Ca/Si ratio 1.0 is gradually decreasing with 35.55~53.59% of water content, and while the fluidity is increasing with the increase of water contents (Figure 2d), the changing laws of which are consistent with that of widely used cement based slurry [24]. Therefore, when the initial Ca/Si ratio is 1.0, the paste can still be adjusted to meet the property requirement of backfill grouting material (close to/higher than 200 mm in fluidity, and higher than 0.6 MPa in 7 d and 28 d UCS [19,23]) by changing the water content.

In summary, these backfill pastes with initial Ca/Si ratio close to 1.0 obtain higher 7 d UCS and better solidification for an SR-NS system and a SR-FFA-NS system. However, the mass mixing proportion of the backfill pastes should be further optimized to meet the performance requirement and reduce the cost owing to the excessive NS (such as initial Ca/Si ratio lower than 1.0 and pan-alkali on surface).

### 3.2. The Proposed BHG Alternative to NS

Considering the economy and engineering properties, it is urgent to reduce the cost and improve the property of SR-FFA based geopolymer backfill material. Generally, there are two ways to explore: (a) directly reduce the addition of NS in the SR-FFA-NS system paste, and (b) employ another low-cost alkaline activator to partially replace expensive NS. In the method (a), the less addition of NS leads directly to lower pH value and lower later strength, performing worse solidification of backfill paste. However, the method (b) that was used can obtain a better effect in terms of solidification and later strength attributed to the double-activation from used activators. Therefore, it is significant to explore another activator alternative to NS to prepare the SR-FFA based geopolymer backfill paste.

It was known that the inorganic salts CaCl_2_, Ca(OH)_2_ and CaSO_4_ in SR react with NS [24]. Here, the inspiration is derived from the soluble inorganic salts in SR. Firstly, the expensive chemical reagents CaCl_2_, CaSO_4_ or Ca(OH)_2_ used are unrealistic for preparing the backfill paste. Then, based on the excessive NS and the lower initial Ca/Si ratio, available beta-hemihydrate gypsum (BHG) powder composed of CaSO_4_·0.5H_2_O can be explored to investigate the feasibility and effectiveness of alternatives to NS, where the reaction mechanism is not greatly influenced due to no complex additives. Moreover, there are the unpredictable results for BHG alternative to NS on whether BHG improves the macro and micro performance of the fresh and hardened paste. Therefore, it is significant to investigate the specific reaction mechanism and unpredictable performance of SR-FFA based geopolymer backfill paste incorporating BHG.

### 3.3. Influence of BHG Alternative Ratios on Workability and UCS of Backfill Paste

**Figure 3:**
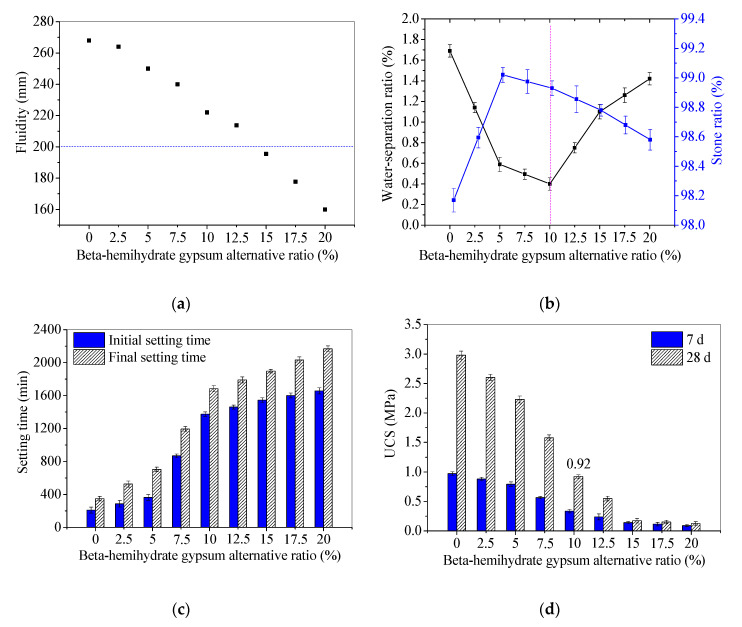
Fresh and mechanical properties of backfill pastes with different BHG alternative to NS: (**a**) fluidities; (**b**) stone ratios and water-separation ratios; (**c**) setting times; (**d**) 7 d and 28 d UCSs.

After determining the method of a BHG alternative to NS, the influence of the BHG alternative ratio was investigated on the workability and UCS of SR-FFA based geopolymer backfill paste. The tested fresh and mechanical properties (including stone ratio, water-separation ratio, initial setting time, final setting time, fluidity and UCS at 7d and 28 d) are shown in Figure 3. The performance requirements needed for the backfill are presented in Table 6.

The tested fluidities of backfill pastes with identical method and conditions are shown in Figure 3a. It can be seen that the fluidities decrease with the increase of BHG alternative ratios 0~20%. Generally, the fluidity is regarded as the best at approximate 200 mm for the backfill materials [21]. When the BHG alternative ratio reaches 10~15%, the fluidities of 196~222 mm are closer to the required workability. Here, the physical water absorption and surface activity of solid powders are the key factors affecting the fluidity [24].

Moreover, the lower water-separation ratio (0.41%) and the higher stone ratio (98.93%) appear at 10% of BHG alternative ratios (Figure 3b). The stone ratios of the SR-FFA-BHG-NS system backfill pastes range from 98.6% to 99.0%, which are closer to 96.7~99.4% of SR-FFA-NS system pastes [24] and higher than 91% of OPC paste prepared by Sun [34]. In addition, the water-separation ratios of SR-FFA-BHG-NS system pastes range from 0.4% to 1.42%, which are closer to 0.14~2.16% of SR-FFA-NS system pastes [24] and particularly lower than 7.25% of OPC paste. Thus, it is presented that the fresh mixtures of SR, FFA, BHG and NSS can be solidified well and cemented for goaf backfill.

Additionally, the setting times are decreasing with the increasing BHG alternative ratio (Figure 3c). The prepared paste with 10% BHG alternative ratios possesses the initial setting time of 1375 min (more than 12 h) and the final setting time of 1683 min (less than 36 h), which satisfies the performance requirement of goaf grouting well [24].

For the mechanical properties, the 7 d and 28 d UCSs also decrease with the increasing BHG alternative ratio (Figure 3d). Generally, the 28 d UCS of backfill material should be higher than 0.6 MPa according to the literatures [19,33]. Even though 7 d UCS of the paste with 10% BHG alternative ratios reaches 0.34 MPa, the 28 d UCS reaches 0.92 MPa that is higher than the required strength. The increase of BHG in the mix (replacing partially sodium silicate) indeed decreases the 28 d unconfined compressive strength (UCS), which reaches 0.92 MPa with 10% BHG alternative ratios according to the required properties for goaf backfill materials (in Table 6). Even though the UCS of 0.92 MPa is not a high value, it is still acceptable for goaf backfill materials owing to the 28 d UCS higher than 0.6 MPa.

Therefore, considering the backfill requirement, the SR-FFA-BHG-NS system paste with 10% BHG alternative ratios (100 g SR, 150 g FFA, 16.24 g CS, 146.16 g NS and 220 g water by mass) obtains the optimal workability and mechanical strength, which corresponds to the initial Ca/Si ratio 1.05 (close to 1.0). The addition of NS is successfully reduced from the original 26.53% to 23.11% coupled with the proper backfill engineering properties. For the cost-reduction of backfill paste, it is calculated that there will be less material cost up to 396.72~560.88 yuan/ton for the SR-FFA-BHG-NS system paste as the additional contents and selling price of NS.

### 3.4. Microstructural Characterization of Backfill Paste and Control

After clarifying the optimal mixing ratio of SR-FFA-BHG-NS system paste as backfill material, the microstructure, mineral phase and product composition were investigated to explain the reaction mechanism. Thus, the pastes without BHG (C0) and with 10% BHG (C4) were used to detect the micromorphology. Furthermore, raw materials, control C0 and paste C4 were utilized to detect the mineral compositions and the gel products at 7 d and 28 d.

#### 3.4.1. Micromorphology for the Product through Scanning Electron Microscopy (SEM)

**Figure 4:**
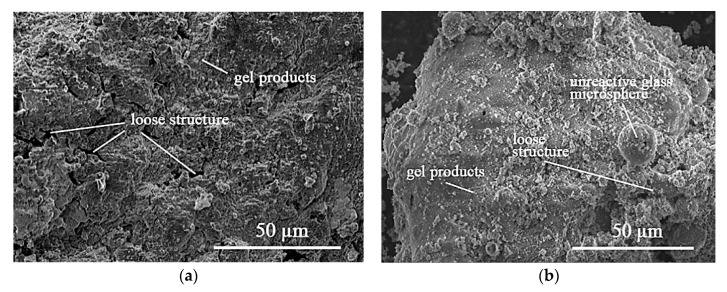
SEM photos of (**a**) control C0 and (**b**) paste C4 cured for 28 days.

To further detect the micromorphology in optimal backfill paste, the control C0 and paste C4 at 28 d were tested by SEM (Figure 4). The purpose of Figure 4 is to observe the cementation and solidification from micromorphology and microstructure, and to analyze the influence of the hydration reaction of BHG on the cementation and solidification of backfill paste. This helps in making some predictions about the property information in gel products.

In general, the cementation of backfill paste determines the microstructure. Control C0 has a denser microstructure, and while paste C4 possesses a looser structure due to the increasing BHG and decreasing NS (Figure 4a). Also, there are small unreactive glass microspheres of FFA in control C0 due to the high reaction degree of alkali-activation, but there are still distinct unreactive glass microspheres on the surface of samples C4 owing to the lower reaction degree of alkali-activation (Figure 4b). Even though paste C4 obtains the loose structure, it has little influence on the application in backfill grouting engineering.

As for the control C0 of the SR-FFA-NS system, previous study explained that the Ca(OH)_2_, CaCl_2_ and CaSO_4_ in SR first react with NS to generate the C-S-H gels at early term [24]. Meanwhile, some FFA particles are also dissolved by alkali-activation to produce the (N,C)–A–S–H gels as the curing time, which is consistent with that of alkali-activated fly ash geopolymer [18]. Therefore, the gel products in paste C0 are the mixtures of C–S–H and (N,C)-A-S-H gels at 28 d. As for the paste C4 of SR-FFA-BHG-NS system, the hydration reaction of BHG immediately occur due to the contact of BHG and water, where the CaSO_4_·0.5H_2_O is changed into CaSO_4_·2H_2_O. The reaction process can be expressed as follows (6):

CaSO_4_·0.5H_2_O + 1.5H_2_O → CaSO_4_·2H_2_O (6)

In summary, it can be concluded that there are distinct unreactive glass microspheres and loose microstructures for optimal paste C4 owing to the lower reaction degree of alkali activation. The cementation and solidification of SR-FFA-BHG-NS system backfill paste are indeed affected by the hydration reaction of BHG and the formation of CaSO_4_·2H_2_O. However, the real changes in gel products need further research by other analysis.

#### 3.4.2. Mineral Phase Analysis through X-Ray Diffraction (XRD)

**Figure 5:**
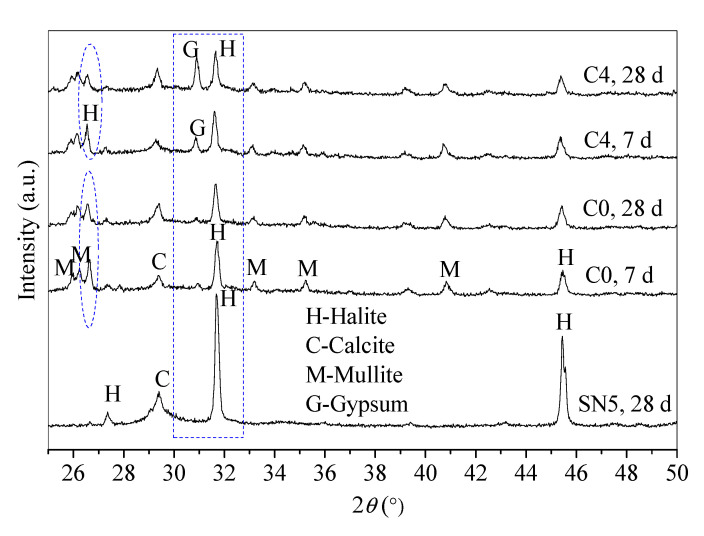
Mineral phases of control C0 (SR-FFA-NS system) and paste C4 (SR-FFA-BHG-NS system) at 7 d and 28 d.

The crystal state of gel products affects the mechanical and microstructural properties [35]. The XRD patterns of control C0 and paste C4 were tested to analyze the effect of BHG alternatives on the mineral phases, as shown in Figure 5.

According to the reaction processes of SR-FFA-NS system paste, amorphous C-S-H gels in SR-NS system paste are produced from the reaction of SR and NS due to there being no detected diffraction peaks of C–S–H products [24]. Compared with XRD patterns of FFA and SR (Figure 1), there is the coexistence of C–S–H gels and (N,C)–A–S–H gels in SR-FFA-NS system paste at 28 d-age, both of which are amorphous. Thereafter, 10% BHG as alternatives are added into SR-FFA-NS system paste, which shows little difference in amorphous gel products at 28 d, and but the crystallinities of gypsum crystals distinctly increase due to higher peak intensity at approximate 31° 2θ. Moreover, the crystallinities of halite crystals decrease at 31~32° 2θ and 26~27° 2θ, which is attributed to more Na^+^ consumptions in the formation of (N,C)-A-S-H gels. Mostly, there is a distinct change in the diffraction peak intensity of gypsum and halite crystals as the curing age from 7 d to 28 d due to the gradual hydration of BHG. However, there are no ettringite crystals detected in control C0 and paste C4, which may result from the dissolved aluminum oxide tetrahedron from FFA preferring to produce the aluminosilicate gels but ettringites at early term. Here, the lack of ettringite peaks is also consistent with previous studies [36]. This may because that the ettringite is destabilized in the presence of Cl^−^ ions [36].

From the XRD patterns, there are no other new substances generated between control C0 and paste C4, which show a similar reaction mechanism except for the hydration of BHG. Therefore, the hydration product of BHG along with generated C-S-H and (N,C)-A-S-H gels determine the final properties of hardened SR-FA based geopolymer backfill paste (C4) with 10% BHG alternative to NS.

#### 3.4.3. Chemical Bonds Analysis for Gel Product through Fourier Transform Infrared (FTIR) Spectroscopy

**Figure 6:**
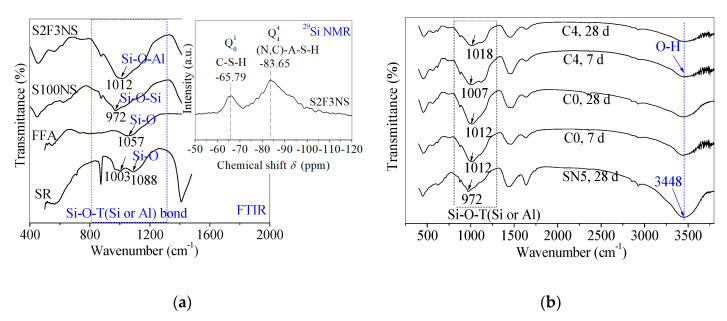
(**a**) Fourier transform infrared (FTIR) spectra of FFA, SR, control S100NS and paste S2F3NS at 180 d, and ^29^Si NMR spectrum of paste S2F3NS at 180 d from reference [24]. (**b**) Chemical bonds analysis of control C0 (SR-FFA-NS system) and paste C4 (SR-FFA-BHG-NS system) at 7 d and 28 d through FTIR spectra.

The FTIR tests were used to further study the reaction changes in chemical bonds for control and backfill paste. In the previous study, the FTIR spectra and ^29^Si nuclear magnetic resonance (NMR) pattern of SR, FFA, control S100NS and paste S2F3NS were tested to detect the gel products (Figure 6a) in the reference [24]. Here, the FTIR spectra of control SN5, control C0 and paste C4 at 7 d and 28 d were measured to clarify the effects of BHG on the gel products in chemical bonds, and verify the correctness of the reaction mechanism, as shown in Figure 6b.

The main bands attributed to Si-O-T (Si or Al) with asymmetrical stretching vibrations are assigned in the 972~1018 cm^−1^ region [32,37]. Compared with control SN5, the chain length of the Si–O–Si bond of control C0 at 28 d increases due to the addition of FFA, where the strong absorption peak in C0 shifts to the higher wavenumber (from 972 cm^−1^ to 1012 cm^−1^). In a previous study, it was proved that SN5 (like control S100NS) promoted the polymerization degree of Si–O–Al bonds in control C0 (like paste S2F3NS), which was attributed to the band of Si–O–T (Si or Al) asymmetrical stretching vibration in FFA shifting to lower wavenumber (from 1057 cm^−1^ to 1012 cm^−1^) [24]. In particular, it worth emphasizing that there is little difference in the chemical bonds in control C0 from 7 d to 28 d, which demonstrates that identical products have been produced at the early term.

In addition, after incorporating 10% BHG alternatives, there is a dispersity for the broadband at 972–1088 cm^−1^ attributed to paste C4, which presents the structural heterogeneity from the complex composition. The structural heterogeneity may result from the existence of CaSO_4_·2H_2_O generated from the hydration of BHG, besides the formation of C-S-H gels and (N,C)-A-S-H gels. However, there is an increase in chain length of the Si–O–Si bond for paste C4 from 7 d to 28 d, and it can be seen that the band of Si–O–Si asymmetrical stretching vibration in C0 shifts to higher wavenumber (from 1007 cm^−1^ to 1018 cm^−1^). It is emphasized that there are identical gel products to the mixtures of C–S–H and (N,C)–A–S–H gels for control C0 and paste C4 at 28 d. In particular, the main bands attributed to O–H bonds in free water are assigned at 3448 cm^−1^, and the absorption peak intensities decrease with the addition of 10% BHG, which also indicates that CaSO_4_·0.5H_2_O in BHG consumes some water to generate CaSO_4_·2H_2_O through the hydration. Therefore, the main gel products of hardened paste C4 are verified as the mixtures of C–S–H gels, (N,C)-A-S-H gels as well as CaSO_4_·2H_2_O. The reaction mechanisms about the formulae (1)~(6) are also proved through FTIR studies.

## 4. Conclusions

To evaluate the application of the beta-hemihydrate gypsum (BHG) alternative to sodium silicate (NS) in SR-FFA-BHG-NS system geopolymer paste as goaf backfill material, a preparation and optimization method was proposed based on the reaction mechanism of the SR-FFA-NS system; and microstructures and gel products of optimal backfill paste with BHG were characterized. Furthermore, the main reaction mechanism was clarified for SR-FFA-BHG-NS system geopolymer backfill paste. The main conclusions drawn are as follows:

(1) The backfill pastes with initial Ca/Si ratio close to 1.0 possess the higher 7 d UCS and better solidification for the SR-NS system and SR-FFA-NS system.
(2) The optimization method of BHG alternative to partial NS is proposed to improve the fresh and mechanical properties of geopolymer backfill pastes based on the reaction mechanism of SR-FFA-NS system.
(3) The optimal backfill paste C4 is prepared with the mixing ratios (100 g SR, 150 g FFA, 16.24 g BHG, 146.16 g NS and 220 g water by mass), which possesses high workability (196–222 mm in fluidity), proper 28 d UCS (0.92 MPa), proper setting time (initial setting time of 1375 min and final setting time of 1683 min) as well as high stone ratio (98.6%). The optimal paste is composed of 10% BHG alternative ratios to NS as well as initial Ca/Si ratio 1.05.
(4) The gel products of paste C4 are determined as the coexistence of C-S-H gel, (N,C)-A-S-H gel and CaSO_4_·2H_2_O. The hydration of CaSO_4_·0.5H_2_O in BHG and the formed C-S-H gel from the reaction of SR and NS determine the workability and UCS at the early term. The (N,C)-A-S-H gels formed from the alkali-activated FFA promote long-term strength development.

In summary, the research results not only propose the optimization method of BHG alternative to partial NS, but also provide significant information about preparation and gel products in the backfill paste. Although the addition of NS is reduced from an original 26.53% to 23.11% (material cost-reduction of 396.72~560.88 yuan/ton), the research results provide the experimental idea and theoretical basis for the utilization of SR, FFA and BHG as backfill material. In this paper, there are two proposed means to achieve the cost-reduction (including the low-cost alkaline activator alternative to partial NS, and directly by the addition-reduction of NS). However, to promote the engineering application of the low-cost and superior performance backfill paste, it is necessary to further explore the other low-cost alkaline activator (such as sulfate salts, silicate salts, and evenly solid waste desulfurization gypsum, etc.) in order to partially or totally replace expensive NS. In the future, there is a need to further evaluate the durability and stability at long curing age as well as the economic cost of the cleaner backfill material prepared using solid wastes.

## Figures and Tables

**Table 1:** Chemical compositions of soda residue (SR) and low-calcium fly ash (FFA) [24].

SR	Percentage (wt.%)	FFA	Percentage (wt.%)
CaCO_3_	64.0	SiO_2_	48.37
Ca(OH)_2_	10.0	Al_2_O_3_	28.90
CaCl_2_	6.0	Fe_2_O_3_	7.00
NaCl	4.0	CaO	4.47
CaSO_4_	3.0	FeO	2.31
SiO_2_	3.0	MgO	1.30
Al_2_O_3_	2.0	LOI ^1^	5.95
Acid insoluble	8.0	Others	1.70

^1^ LOI is the loss on ignition at 1000 °C (wt.%).

**Table 2:** Physical indexes of raw material SR and FFA [24].

Materials	Amount Passing #325 Sieve	Specific Gravity	Specific Surface Area (m^2^/kg)	pH Value at *w*_100_^2^
FFA	77%	2.43	509	8.50
SR	—	2.35	—	8.35

^2^*w*_100_ shows pH values with 100% water content at room temperature.

**Table 3:** Mass mixing proportions of backfill paste about the influence of Initial Ca/Si ratio on 7 d unconfined compressive strength (UCS). SR-soda residue; NS-sodium silicate, and FFA-fly ash.

No.	SR (g)	NS (g)	Water (g)	FFA (g)	Initial Ca/Si Ratio ^3^
SN1	99.53	150.71	120.0	0	0.80
SN2	105.75	150.71	120.0	0	0.85
SN3	111.97	150.71	120.0	0	0.90
SN4	118.49	150.71	120.0	0	0.95
SN5	125.00	150.71	120.0	0	1.00
SN6	130.93	150.71	120.0	0	1.05
SN7	136.85	150.71	120.0	0	1.10
SN8	143.08	150.71	120.0	0	1.15
SN9	149.30	150.71	120.0	0	1.20
SNF1	99.53	150.71	180.0	90	0.80
SNF2	105.75	150.71	180.0	90	0.85
SNF3	111.97	150.71	180.0	90	0.90
SNF4	118.49	150.71	180.0	90	0.95
SNF5	125.00	150.71	180.0	90	1.00
SNF6	130.93	150.71	180.0	90	1.05
SNF7	136.85	150.71	180.0	90	1.10
SNF8	143.08	150.71	180.0	90	1.15
SNF9	149.30	150.71	180.0	90	1.20

^3^ Initial Ca/Si ratio means the molar ratio of CaCl_2_, Ca (OH)_2_ and CaSO_4_ in SR to NS.

**Table 4:** Mass mixing proportions of backfill paste about the influence of water content on 7 d UCS.

No.	SR (g)	NS (g)	Water (g)	FFA (g)	Initial Ca/Si Ratio	Water Content (%)
W1	125.00	150.71	130.0	90	1.00	35.55
W2	125.00	150.71	140.0	90	1.00	38.28
W3	125.00	150.71	150.0	90	1.00	41.02
W4	125.00	150.71	160.0	90	1.00	43.75
W5	125.00	150.71	170.0	90	1.00	46.48
W6	125.00	150.71	186.4	90	1.00	50.97
W7	125.00	150.71	196.0	90	1.00	53.59

**Table 5:** Mass mixing proportions of backfill paste activated by beta-hemihydrate gypsum (BHG) alternative to partial sodium silicate (NS).

No.	SR (g)	FFA (g)	BHG (g)	NS (g)	Alternative Ratio (%)	Water (g)	Initial Ca/Si Ratio
C0	100	150	0.00	162.40	0.0	220	0.75
C1	100	150	4.06	158.34	2.5	220	0.82
C2	100	150	8.12	154.28	5.0	220	0.89
C3	100	150	12.18	150.22	7.5	220	0.97
C4	100	150	16.24	146.16	10.0	220	1.05
C5	100	150	20.30	142.10	12.5	220	1.13
C6	100	150	24.36	138.04	15.0	220	1.22
C7	100	150	28.42	133.98	17.5	220	1.32
C8	100	150	32.48	129.92	20.0	220	1.42

**Table 6:** The performance requirements of the grosuting material for goaf backfill [19,21,22,23,24,33,34].

Main Properties	Fluidity	28 d UCS	Initial Setting Time	Final Setting Time	Stone Ratio
Required value	≥200 mm	>0.6 MPa	≥12 h	≤36 h	>91%
Paste C4	196 mm	0.92 MPa	1375 min	1683 min	98.93%

## References

[1] Tho-In T., Sata V., Boonserm K., Chindaprasirt P. (2018). Compressive strength and microstructure analysis of geopolymer paste using waste glass powder and fly ash. J. Clean. Prod..

[2] Zhuang X.Y., Chen L., Komarneni S., Zhou C.H., Tong D.S., Yang H.M., Yu W.H., Wang H. (2016). Fly ash-based geopolymer: Clean production, properties and applications. J. Clean. Prod..

[3] Zhao X.H., Liu C.Y., Wang L., Zuo L.M., Zhu Q., Ma W. (2019). Physical and mechanical properties and micro characteristics of fly ash-based geopolymers incorporating soda residue. Cem. Concr. Compos..

[4] Zhao X., Liu C., Zuo L., Zhu Q., Ma W., Liu Y. (2020). Preparation and characterization of press-formed fly ash cement incorporating soda residue. Mater. Lett..

[5] Zhang J., Tan H., He X., Yang W., Deng X. (2020). Utilization of carbide slag-granulated blast furnace slag system by wet grinding as low carbon cementitious materials. Constr. Build. Mater..

[6] Castel A., Foster S.J. (2015). Bond strength between blended slag and Class F fly ash geopolymer concrete with steel reinforcement. Cem. Concr. Res..

[7] Sarker P.K., McBeath S. (2015). Fire endurance of steel reinforced fly ash geopolymer concrete elements. Constr. Build. Mater..

[8] Shi C.J., Jimenez A.F., Palomo A. (2011). New cements for the 21st century: The pursuit of an alternative to Portland cement. Cem. Concr. Res..

[9] Nematollahi B., Sanjayan J., Shaikh F.U.A. (2015). Synthesis of heat and ambient cured one-part geopolymer mixes with different grades of sodium silicate. Ceram. Int..

[10] Assi L., Carter K., Deaver E., Anay R., Ziehl P. (2018). Sustainable concrete: Building a greener future. J. Clean. Prod..

[11] Saha S., Rajasekaran C. (2017). Enhancement of the properties of fly ash based geopolymer paste by incorporating ground granulated blast furnace slag. Constr. Build. Mater..

[12] Bernal S.A., de Gutierrez R.M., Provis J.L., Rose V. (2010). Effect of silicate modulus and metakaolin incorporation on the carbonation of alkali silicate-activated slags. Cem. Concr. Res..

[13] Pasupathy K., Berndt M., Sanjayan J., Rajeev P., Cheema D.S. (2017). Durability of low-calcium fly ash based geopolymer concrete culvert in a saline environment. Cem. Concr. Res..

[14] National Development and Reform Commission of China (2011). Implementing Scheme of Mainly Solid Waste Utilization (Beijing).

[15] Shang J., Dai J.G., Zhao T.J., Guo S.Y., Zhang P., Mu B. (2018). Alternation of traditional cement mortars using fly ash-based geopolymer mortars modified by slag. J. Clean. Prod..

[16] Liu Y.L., Wang Y.S., Fang G.H., Alrefaei Y., Dong B.Q., Xing F. (2018). A preliminary study on capsule-based self-healing grouting materials for grouted splice sleeve connection. Constr. Build. Mater..

[17] Chindaprasirt P., Rattanasak U. (2017). Characterization of the high-calcium fly ash geopolymer mortar with hot-weather curing systems for sustainable application. Adv. Powder Technol..

[18] Somna K., Jaturapitakkul C., Kajitvichyanukul P., Chindaprasirt P. (2011). NaOH-activated ground fly ash geopolymer cured at ambient temperature. Fuel.

[19] Li H., Wu A.X., Wang H.J. (2019). Evaluation of short-term strength development of cemented backfill with varying sulphide contents and the use of additives. J. Environ. Manag..

[20] Hadi M.N.S., Al-Azzawi M., Yu T. (2018). Effects of fly ash characteristics and alkaline activator components on compressive strength of fly ash-based geopolymer mortar. Constr. Build. Mater..

[21] Sun Q., Tian S., Sun Q.W., Li B., Cai C., Xia Y.J., Wei X., Mu Q.W. (2019). Preparation and microstructure of fly ash geopolymer paste backfill material. J. Clean. Prod..

[22] Zhou B., Wang L., Ma G., Zhao X., Zhao X. (2020). Preparation and properties of bio-geopolymer composites with waste cotton stalk materials. J. Clean. Prod..

[23] Zhao X.H., Liu C.Y., Zuo L.M., Wang L., Zhu Q., Wang M.K. (2019). Investigation into the effect of calcium on the existence form of geopolymerized gel product of fly ash based geopolymers. Cem. Concr. Compos..

[24] Zhao X., Liu C., Zuo L., Wang L., Zhu Q., Liu Y., Zhou B. (2020). Synthesis and characterization of fly ash geopolymer paste for goaf backfill: Reuse of soda residue. J. Clean. Prod..

[25] Yu S.J., Bi W.Y., Zheng L.G., Duan Y.L. (2007). Impact of Cl- on environment when industrial soda residue is applied in highway project. Prog. Environ. Sci. Technol..

[26] Gorhan G., Kurklu G. (2014). The influence of the NaOH solution on the properties of the fly ash-based geopolymer mortar cured at different temperatures. Compos. Part B Eng..

[27] Pavithra P., Reddy M.S., Dinakar P., Rao B.H., Satpathy B.K., Mohanty A.N. (2016). A mix design procedure for geopolymer concrete with fly ash. J. Clean. Prod..

[28] Ouattara D., Belem T., Mbonimpa M., Yahia A. (2018). Effect of superplasticizers on the consistency and unconfined compressive strength of cemented paste backfills. Constr. Build. Mater..

[29] Yousefi Oderji S., Chen B., Ahmad M.R., Shah S.F.A. (2019). Fresh and hardened properties of one-part fly ash-based geopolymer binders cured at room temperature: Effect of slag and alkali activators. J. Clean. Prod..

[30] Niroshan N., Sivakugan N., Veenstra R.L. (2017). Laboratory study on strength development in cemented paste backfills. J. Mater. Civil Eng..

[31] Fahim Huseien G., Mirza J., Ismail M., Ghoshal S.K., Abdulameer Hussein A. (2017). Geopolymer mortars as sustainable repair material: A comprehensive review. Renew. Sust. Energ. Rev..

[32] Swanepoel J.C., Strydom C.A. (2002). Utilisation of fly ash in a geopolymeric material. Appl. Geochem..

[33] Li B.J., Li G.Z. (2011). Study on mechanical properties of soda residue/fly ash composite cementitious material. Adv. Mater. Res. Switz..

[34] Sun Q. (2009). Experimental Study on the Properties of Fly Ash-Based Grouting Backfill Materials. Master‘s Thesis.

[35] Ye N., Yang J.K., Ke X.Y., Zhu J., Li Y.L., Xiang C., Wang H.B., Li L., Xiao B. (2014). Synthesis and characterization of geopolymer from bayer red mud with thermal pretreatment. J. Am. Ceram. Soc..

[36] Lin Y., Xu D., Zhao X. (2020). Effect of soda residue addition and its chemical composition on physical properties and hydration products of soda residue-activated slag cementitious materials. Materials.

[37] Garcia-Lodeiro I., Palomo A., Fernandez-Jimenez A., Macphee D.E. (2011). Compatibility studies between N-A-S-H and C-A-S-H gels. Study in the ternary diagram Na_2_O-CaO-Al_2_O_3_-SiO_2_-H_2_O. Cem. Concr. Res..

